# Characteristic time in highly motivated movements of children and adults through bottlenecks

**DOI:** 10.1038/s41598-021-84324-4

**Published:** 2021-03-03

**Authors:** Hongliu Li, Jun Zhang, Long Xia, Libing Yang, Weiguo Song, Kwok Kit Richard Yuen

**Affiliations:** 1 State Key Laboratory of Fire Science, University of Science and Technology of China, Jinzhai Road 96, Hefei, Anhui People’s Republic of China; 2 Department of Architecture and Civil Engineering, City University of Hong Kong, Kowloon, 999077 Hong Kong People’s Republic of China; 3 College of Civil Engineering and Architecture, Hunan Institute of Science and Technology, Xueyuan Road, Yueyang, Hunan People’s Republic of China

**Keywords:** Civil engineering, Fluid dynamics, Complex networks, Nonlinear phenomena

## Abstract

Current codes for fire protection of buildings are mainly based on the movement of adults and neglect the movement characteristic of pre-school children. Having a profound comprehension of the difference between children and adults passing bottlenecks is of great help to improve the safety levels of preschool children. This paper presents an experimental study on the bottleneck flow of pre-school children in a room. The movement characteristics of children’s and adults’ bottleneck flow are investigated with two macroscopic properties: density and speed profiles as well as microscopic characteristic time: motion activation time, relaxation time, exit travel time and time gap. Arch-like density distributions are observed both for highly motivated children and adults, while the distance between the peak density region and the exit location is shorter for children and longer for adults. Children’s movement is less flexible manifested as longer motion activation time and longer relaxation time compared to that of adults. The findings from this study could enhance the understanding of crowd dynamics among the children population and provide supports for the scientific building design for children’s facilities.

## Introduction

Children are a common sight in kindergartens and public places like museums, shopping malls and amusement parks. A sad but recurring reminder of the importance of children’s movement studies is severe accidents such as the 2018 Kemerove fire in Russia^1^, the 2001 Nanchang Nursery Fire in China^2^, and the 1999 Sealand Youth Training Center Fire in South Korea^3^. With the expanded coverage of preschool education^4^ and children’s play places, children are not always under one-on-one supervision. They have to move in groups under the command of tutors or safety staff in emergencies. However, current codes for fire protection design of the building are mainly based on the movement characteristics of adults and there are few considerations on that of pre-school children. To design comfortable and safe infrastructures for children, it is urgent to have a better understanding and quantification of children’s evacuation characteristics.

Bottleneck as a common geometry in many pedestrian infrastructures often restricts the pedestrian flow^4–11^ and the arch-like distributions of pedestrians around an exit are observed both in adults and children groups when they are in a rush^4,8,10^. It is reported that the peak density region locates in the front of the bottleneck and the distance to the entrance is related to the type of pedestrians (running pre-school children: 0.3 m, walking adults: 1.0 m)^4,12^. Pedestrian congestion phenomenon is observed during the pickup period in simulation^13,14^ and it affects the evacuation efficiency. Queuing in front of the door helps social groups of children evacuate compared to gather around the exit broadly^15^. However, we have a few understandings of the density distribution difference of children and adults in highly motivated running conditions. Besides, crowd velocity is a key factor influencing the pedestrian flow. Walking speeds of pre-school children are smaller than that of pupils^16^ and adults^17^, while preschool children tend to run^16^ and they move faster than adults at the same density^18^ in evacuation drills. Focusing on the movement of adults, the higher the speed is, the more likely the movement direction pointing to the exit is based on the previous empirical study^8^. Considering previous velocity characteristics, both pedestrian type (adults or children) and movement motivation (walking or running) show an impact on the velocity of pedestrians. This inspires us to further quantify the difference of the velocity (both the value and the direction) between highly-motivated preschool children and adults passing bottlenecks.

In addition to the above macro factors influencing pedestrians’ movement, characteristic time as the factor depicting the microscopic motion characteristics of pedestrians are more susceptible to neglect. Relaxation time is defined to represent the capability of an individual to adjust the velocity^10^ and empirical studies quantify it around 0.6 s^19,20^ in walking conditions. With the increasing angular difference in turning movement, relaxation time shows a growth trend^19^. However, as previous researches about relaxation time focus on the walking conditions, we redefine the relaxation time to quantify the speed adjustment ability for highly motivated pedestrians during the running condition and compare the difference between that of children and adults.

Besides the whole movement process, the start-up stage of movements is investigated to study the reaction ability of pedestrians. The pre-evacuation time is the time interval from the given alarm to the first deliberate evacuation movement. Previous studies point out that preschool children spend longer pre-evacuation time compared to that of adults^21,22^. This phenomenon inspires us to define a new characteristic time—motion activation time to quantify the capacity of pedestrians to start moving when they have enough space to move.

Moreover, as people tend to gather around the exit and form high-density regions in previous bottleneck experiments^4,8,12,23^, to quantify the magnitude of clogging events, time headway, the time interval of two consecutive pedestrians passing through exits, are investigated. Previous studies propose that higher competition leads to longer time headway both for children and for adults^4,24^. However, there exists a great difference in movement motivation of previous researches^4,24^, which doubts us the difference of time gap between children and adults both in similar high motivation. Considering this, we aim to study the distribution of the time gap of highly motivated children and adults.

To date, there is still a lack of highly motivated children’s movement characteristics based on a controlled experiment. Besides, current researches mainly focus on the macroscopic properties (like density, speed, and flow), the microscopic properties of children are less examined. Moreover, a quantitative comparison of movement characteristics between highly-motivated children and adults passing bottlenecks is still absent. Therefore, to narrow this research gap, it is important to glean insights from the bottleneck flow characteristics of children and get the difference between that of adults.

In this study, a laboratory-controlled bottleneck experiment in a kindergarten is carried out to understand the movement and behavioral characteristics of children. Density and velocity describe the children’s movements and micro-motion characteristics are evaluated from the beginning to the end of the movement. Meanwhile, the comparison study reveals the differences between that of children and adults. The main contribution and novelty of our work is a direct comparison of children’s and adults’ characteristic time discussed above during the movement process applying the empirical method.

The rest of the paper is organized as follows. In “Method” section, a detailed introduction of the experiment and the measurement methods applied in this study are presented. In “Experimental profiles” section presents the experimental profiles of density and speed compared between children and adults. In “Characteristic time” section shows the characteristic time during the whole experimental process compared between children and adults. The concluding remarks are made in Summary” section.

## Method

### Experimental setup

**Figure 1:**
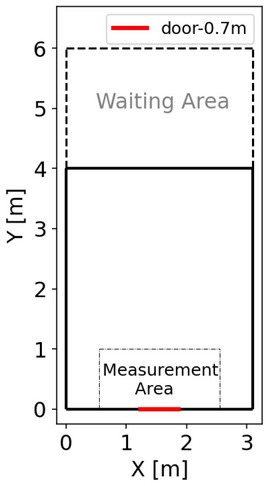
The sketch of the experiment. The exit width is fixed as 0.7 m. The measurement area (2 m × 1 m) is selected to measure the time evolution of density and speed in “Time profiles of density and speed” section. To measure the movement characteristics of pedestrians, the coordinate system takes the lower-left corner as the origin point, the direction parallel to the exit as the X-axis, and the direction perpendicular to the exit as the Y-axis.

The experiment was performed in March 2019 in a kindergarten in Hunan province in China. A total of 40 pre-school children participated in the experiment and they were asked to pass through an artificial room, illustrated in Fig. 1. The boundary of the artificial room was built with plastic security fences (height: 0.75 m) and wooden boards (size: 1.20 m × 1.00 m × 0.05 m). To avoid bruising or worse during the experiment, foam materials were applied to cover the edges of the boards. Two staff and several tires played supporting roles to improve the stability of the boards, avoiding being knocked over by the children. The participated children were asked to leave the room quickly after hearing the command as if in a fire. As fire evacuation drills are conducted in the kindergarten every year, children understood the situation and the command. The participants are around 3–5 years old and the mean height and weight are listed in Table 1. Children were asked to stand in the waiting area located 4.00 m away from the exit and the initial density in the waiting area is 6.45 ped/m^2^. The colored hats wore by each child helped extract the trajectories after the experiment. Three tutors of the kindergarten helped maintain the experimental order and organize the children. They also gave instructions to the children to start each run during the experiment. Children were highly motivated and tried to pass the exit quickly during the whole experiment (see Appendix E for details). A total of 12 sets of valid data are obtained.

**Table 1:** Basic information of participated children in this study.

	Number	Height/m	Weight/kg
Male	23	1.13 ± 0.08	20.07 ± 5.14
Female	17	1.08 ± 0.08	17.74 ± 2.75
Overall	40	1.11 ± 0.08	19.11 ± 4.43

One Sony FDR-AX100 camera (resolution: 1920 × 1080 pixels, frame rate: 25 fps) located on the third floor recorded the whole procedure of the experiment. The trajectories were extracted semi-automatically from video recordings after calibrating the intrinsics and exstrinsics of the camera by PeTrack^25^ software. All subsequent analyses are conducted based on the trajectories combining the recorded videos. No ethical concerns were mentioned in this study. Only anonymous data were used for the studies and the methodological design, data storage process, and the access authorization for data was approved by the ethics committee of the University of Science and Technology of China. We obtained the informed consent from all participants’ legal guardians. All methods were performed in accordance with the relevant guidelines and regulations.

### Data processing method

#### Smoothed trajectories

Due to the technical limitations, we tracked the locations of the heads of pre-school children to obtain the trajectories of participants. The heads of pedestrians were swaying when their body weight shifted from one leg to the other during movement. To eliminate the oscillations caused by the swaying heads, the trajectories are smoothed by averaging the pedestrians’ space coordinate values based on Eq. (1).

1 $$x_{i} = {{\mathop \sum \limits_{{t_{0} = i - 12}}^{{t_{1} = i + 12}} x_{t} } \mathord{\left/ {\vphantom {{\mathop \sum \limits_{{t_{0} = i - 12}}^{{t_{1} = i + 12}} x_{t} } {25}}} \right. \kern-\nulldelimiterspace} {25}},\quad y_{i} = {{\mathop \sum \limits_{{t_{0} = i - 12}}^{{t_{1} = i + 12}} y_{t} } \mathord{\left/ {\vphantom {{\mathop \sum \limits_{{t_{0} = i - 12}}^{{t_{1} = i + 12}} y_{t} } {25}}} \right. \kern-\nulldelimiterspace} {25}}$$

Note that (*x*_*t*_, *y*_*t*_) represents the original coordinates of the child at the time *i* frame. *i* + 12 represents 12 frames after *i* frame. (*x*_*i*_, *y*_*i*_) is the mean value of 25 frames (1 s) original coordinate. To eliminate the interference of swaying on instantaneous angular velocity, we smoothed the trajectories by averaging the participants’ space coordinate values within 1 s. See Appendix B for the comparison between raw and smoothed trajectories.

#### Local density and local speed

The local density and local speed are calculated based on the Gaussian method proposed in^9^. Here we only introduce the method briefly.

The local density $\rho$ at the place $\vec{r} = \left( {x,y} \right)$ at time *t* is measured as:

$$\rho \left( {\vec{r},t} \right) = \mathop \sum \limits_{j} f\left( {\vec{r}_{j} \left( t \right) - \vec{r}} \right),\quad f\left( {\vec{r}_{j} \left( t \right) - \vec{r}} \right) = \frac{1}{{\pi R^{2} }}\exp \left[ {{{ - \left| {\vec{r}_{j} \left( t \right) - \vec{r}} \right|^{2} } \mathord{\left/ {\vphantom {{ - \left| {\vec{r}_{j} \left( t \right) - \vec{r}} \right|^{2} } {R^{2} }}} \right. \kern-\nulldelimiterspace} {R^{2} }}} \right]$$.

The local speed $v$ at the place $\vec{r} = \left( {x,y} \right)$ at time *t* is measured as:

$$\vec{v}\left( {\vec{r},t} \right) = \frac{{\mathop \sum \nolimits_{j} \vec{v}_{j} f\left( {\vec{r}_{j} \left( t \right) - \vec{r}} \right)}}{{\mathop \sum \nolimits_{j} f\left( {\vec{r}_{j} \left( t \right) - \vec{r}} \right)}}$$.

*R* is a measurement parameter. The greater *R* is, the greater the effective measurement radius is.

#### Motion activation time

To study the movement of pedestrians at the beginning of the motion in the experiment, we define a characteristic time as the motion activation time *t*_*MA*_ to measure the time taken from having enough space to start moving. The motion activation time *t*_*MA*_ is defined as Eq. (2).

2 $$\begin{aligned} & d_{m} \left( t \right) = \sqrt {(x_{t} - x_{0} )^{2} + (y_{t} - y_{0} )^{2} } \\ & \exists t, s.t.d_{m} \left( t \right) > d_{mth} \Rightarrow t_{ma} = t \\ & \delta d_{\alpha } \left( t \right) = d_{a} \left( t \right) - d_{{a_{0} }} \\ & \exists t, s.t.\delta d_{\alpha } \left( t \right) > d_{\alpha th} \Rightarrow t_{a} = t \\ & t_{MA} = t_{m} - t_{a} \\ \end{aligned}$$

$t_{MA}$ represents the motion activation time defined in this study. $t_{m}$ represents the time when the Euclidean distance $d_{m}$ between the current position (*x*_*t*_, *y*_*t*_) to the initial position (*x*_*0*_,*y*_*0*_) exceeds a value $d_{mth}$. Note that the initial position refers to the position at the beginning of the experiment.$d_{\alpha 0}$ represents the initial spatial distance. $t_{ath}$ represents the time when the spatial distance $d_{\alpha }$ increases by a certain value $d_{\alpha th}$. Here, based on the step length of adults^26^, $d_{mth}$ is set as 0.2 m and $d_{\alpha th}$ is set as 0.1 m for students. Combined the relation between the shoulder width of pre-school children and that of students (see Appendix A for the data of shoulder width), $d_{mth}$ is set as 0.13 m for pre-school children and $d_{\alpha th}$ is set as 0.065 m for pre-school children in this study.

**Figure 2:**
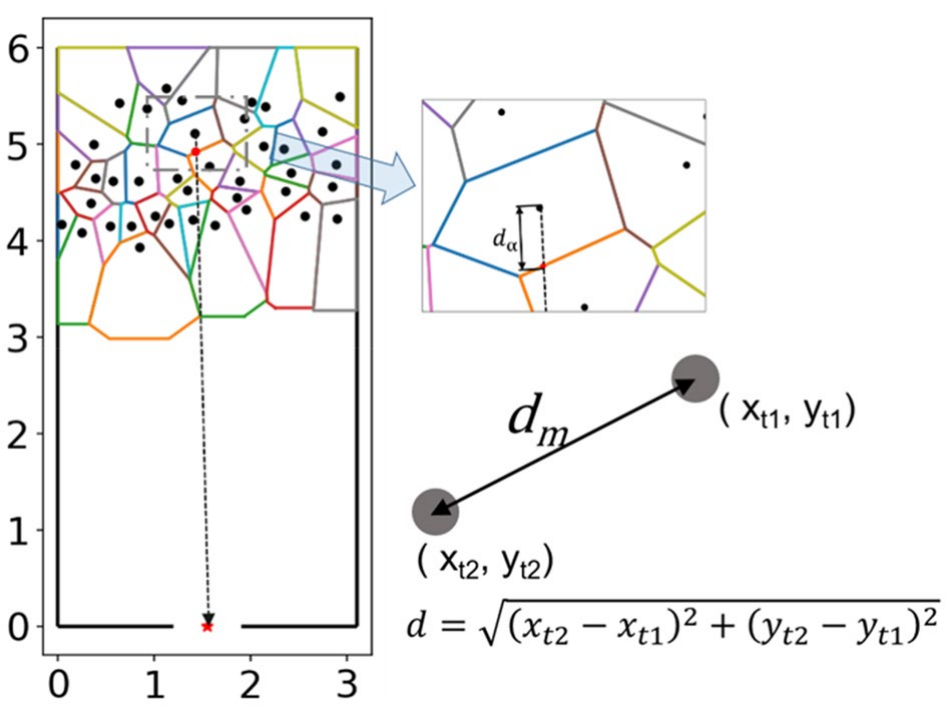
Definition of motion activation time calculation method. Black points represent the positions of each pedestrian in the first frame. Voronoi diagrams represent the space each pedestrian takes up at this time. The red star represents the middle point of the exit and we assume that each pedestrian moves towards the target at the beginning of the experiment. The red point represents the intersection between the moving target and the boundary of the pedestrian’s occupied space. The distance *d*_*a*_ between the pedestrian position and the intersection represents the personal available distance. The grey solid circles represent an individual at two different time in an experiment. $d_{m}$ represents the movement distance calculated by the coordinate of the individual. When $d_{m} > d_{mth}$, the time is set as the moving time *t*_*m*_. When $d_{\alpha } > d_{\alpha \_threshold}$, the time is set as the available movement time $t_{a}$. Motion activation time is obtained by $t_{MA} = t_{m} - t_{a}$ (See Eq. 2).

Voronoi Diagram can be used to define the personal space in pedestrian movement^27^. Considering this, the spatial distance $d_{\alpha }$ related to the personal space is measured based on the Voronoi Diagram based method^28^. JPSReport platform (http://www.jupedsim.org/jpsreport/) is applied to obtain the Voronoi Diagram coordinate of each pedestrian per frame. $d_{m}$ is calculated based on the actual positions from the smoothed trajectories as it is unrelated to the personal space. See Fig. 2 for the detailed description.

#### Relaxation time

To measure the acceleration ability of pedestrians, relaxation time *t*_*re*_ is defined as Eq. (3).

For each pedestrian,

3 $$\begin{aligned} \delta v\left( t \right) & = v_{t + 1} - v_{t} \\ \exists t_{1} ,t_{2} ,s.t. \mathop \sum \limits_{t = t1}^{{t_{2} }} \delta v\left( t \right) & \ge 0.1 \;{\text{m/s}} \Rightarrow t_{re} = t_{2} - t_{1} \\ \end{aligned}$$

$t_{re}$ represents the relaxation time defined in this work. $\delta v\left( t \right)$ represents the speed difference by subtracting the speed of the consecutive two frames. $v_{t + 1}$ for the speed at (*t* + 1) frame and $v_{t}$ for the speed at the time *t* frame. By adding up $\delta v\left( t \right)$ for (*t*_*2*—_*t*_*1*_) frames, if the cumulative sum exceeds a threshold, the time required is counted as relaxation time. In this study, the threshold is set as 0.1 m/s. Only the first time the speed increment exceeds the threshold is counted as the relaxation time.

In this definition, the acceleration time should be continuous. If the deceleration behavior appears and interrupts the acceleration process, the corresponding data point is omitted. For example, while the cumulative sum is smaller than the threshold, if the $\delta v\left( t \right)$ is smaller than 0, the accumulation is interrupted.

#### Travel time

**Figure 3:**
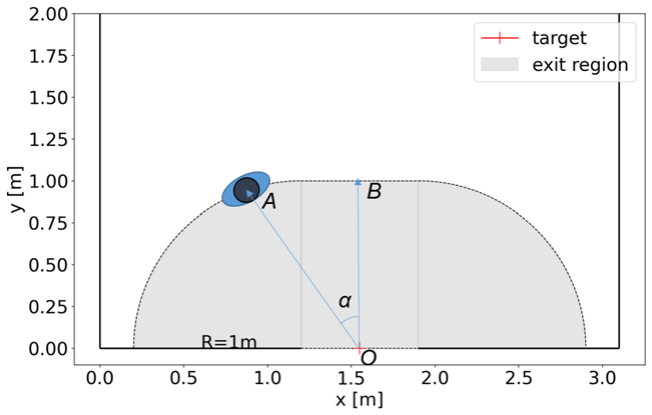
Definition of exit region and incident angle in pre-school children’s experiment. The exit region consists of a rectangle and two 1/4 circles (grey regions). The angle *α* between the vector $\overrightarrow {OA}$ and vector $\overrightarrow {OB}$ represents the incident angle of pedestrians. The angle between $\overrightarrow {OA}$ and $\overrightarrow {OB}$ is defined as incident angle. Point A represents the entering exit region position of the pedestrian.

The exit region is defined to measure the movement characteristics near the exit. Figure 3 shows the definition of the exit region. To make the distance between the pedestrian’s entering position to the exit location similar, the radius of the exit region is *R*, so the exit region consists of two 1/4 circles with a radius of *R* and a rectangle (*R* × *b*). The *R* in pre-school children’s experiment is set as 1 m and the *R* of adults’ experiment is 1.5 m based on the ratio of mean shoulder breadth (see Appendix A for the data of shoulder width).

To investigate the traffic efficiency, travel time *tt*_*exit*_ inside the exit region is defined as the time range from the first time a pedestrian entering the exit region to the time when the pedestrian passes the exit. Incident angle *α* is defined to quantify the impact of the initial position in the process of passing the exit region. The Voronoi density inside the exit region when a pedestrian enters the exit region is considered to measure its influence on the passage efficiency.

## Experimental profiles

In this section, macroscopic parameters of movement characteristics are compared between pre-school children and adults. The temporal and spatial evolution of density and velocity are considered. The 1.05 m width bottleneck experiment of students is chosen to compare with that of pre-school children’s experiment. See Appendix A for the selection reason.

The trajectories of children and adults are averaged to avoid the influence of the swaying heads based on the method proposed in “Smoothed trajectories” section. The raw trajectories and smoothed trajectories of children and adults are illustrated in Appendix A.

### Time profiles of density and speed

**Figure 4:**
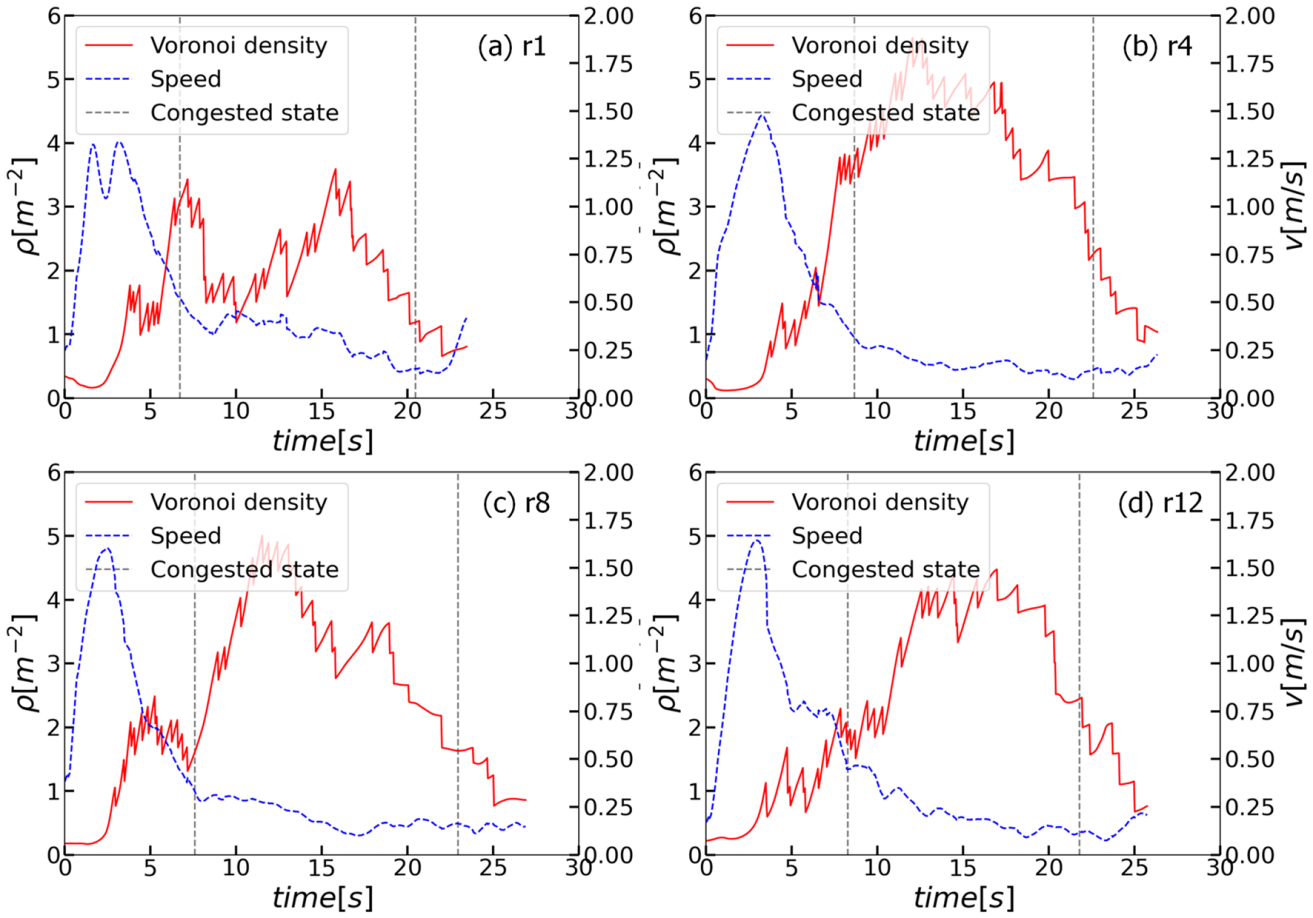
Time profiles of density and speed in the measurement area. (**a**) r1. (**b**) r4. (**c**) r8. (**d**) r12. Red solid lines represent the time profiles of density and blue dashed lines represent the time profiles of speed. The range between grey dashed lines represent the selected congested state. See Appendix C for the time profiles in other experimental runs.

JPSReport (http://www.jupedsim.org/jpsreport/) is applied to obtain the time evolution of Voronoi density and speed in the measurement area (see Fig. 1 for the definition) as shown in Fig. 4. The speed in the measurement area reaches up to 1.50 m/s when the first few children pass the exit. When most of the children reach and gather around the exit, the speeds decrease sharply and fluctuate around 0.25 m/s. Meanwhile, the density increases to 4 ped/m^2^ and remains at a high level for about 10 s. With time, the children gathering around the exit dissipate, resulting in the decreasing density.

To avoid the influence of the transient state on the dynamics of pedestrian experiments, it is necessary to select the congested state of the pedestrian flow. Besides, to avoid the non-reproducible characteristic of the manual selection process, the method defined in^29^ is applied to detect the congested state in the bottleneck experiment automatically. The results of the congested state are shown in Table 2. See Appendix D for the range of density and speed in the congested state.

**Table 2:** Time range of congested state in each run.

Run	1	2	3	4	5	6	7	8	9	10	11	12
Start/frame	167	221	218	216	193	219	211	189	203	182	221	206
End/frame	511	579	525	564	631	601	589	573	604	514	531	544

FPS = 25 frame/s. Note that the congested state is applied in “Density profiles” and “Velocity profiles” sections.

### Density profiles

Density is chosen to measure the degree of a crowd gathering in the experiment during the congested state. The local density calculation method is introduced briefly in “Local density and local speed” section.

**Figure 5:**
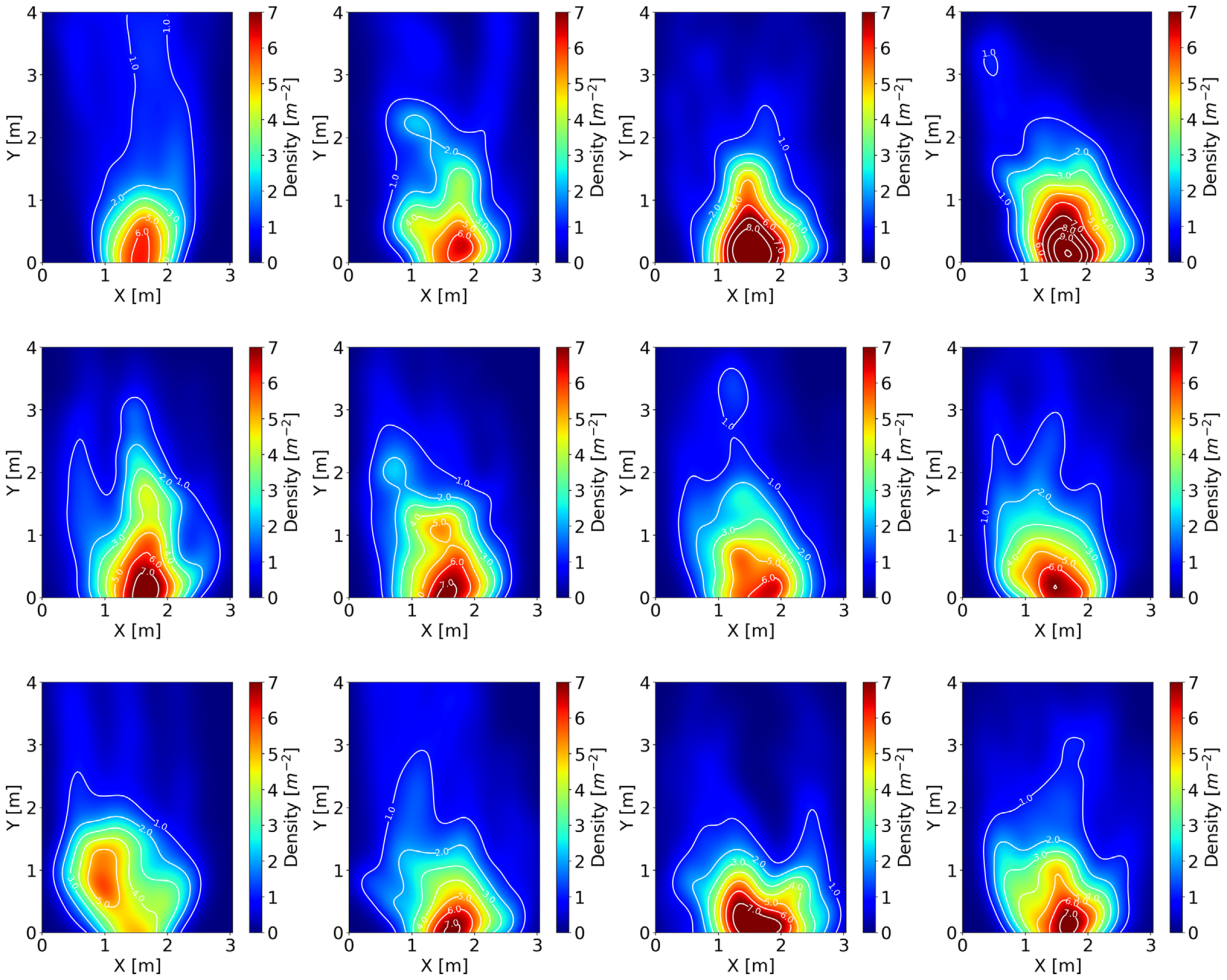
Density profiles during the congested stage in this study. Top row: r1, r2, r3, r4; middle row: r5, r6, r7, r8; bottom row: r9, r10, r11, r12. Color for the density value and white lines for density contours. Noted that in r9, the high-density region locates far from the exit, which is different from other experimental runs. Based on the experimental videos, the phenomenon is due to several children start playing in the region and fail to pass the exit quickly in this experimental run.

Applying the method mentioned above, the density profiles during the congested state are plotted in Fig. 5. It is observed that the distributions of density are inhomogeneous over space and we observe the gradual change from high density to low density. Similar to the previous study of children passing through the bottleneck^4^, the congested areas in front of the exit display an arch-like shape, and the shape of the peak density region is similar to ellipses, whereas the peak density reaches about 7 ped/m^2^ in this study which is a bit smaller than the value 8 ped/m^2^ in the previous study^4^. Moreover, the peak density locates in the middle of the exit, which is closer to the bottleneck entrance compared to the previous study^4^. Focusing on the high motivated adults in the 1.05 m width bottleneck experiment in^8^, the density in the front of the exit region is arch-like distribution and the peak density reaches 6 ped/m^2^ and locates 0.5 m away from the exit entrance. However, compared to the low motivated adults^4,12^, both in 1.0 m width and 1.1 m width bottleneck experiment (bottleneck length is 4.0 m), the distribution of density is teardrop-shaped like and the peak density locates 1.0 m away in front of the bottleneck entrance.

Ultimately, the density distribution around the exit is arch-like for high motivated pedestrians despite the children or adults and teardrop-like for low motivated adults. The peak density appears in the middle and the front of the exit for the bottleneck without length and with length, respectively. Besides, we suspect that increasing bottleneck length has an effect of keeping the peak density region away from the bottleneck entrance. Further empirical study is needed to verify the assumption.

### Velocity profiles

**Figure 6:**
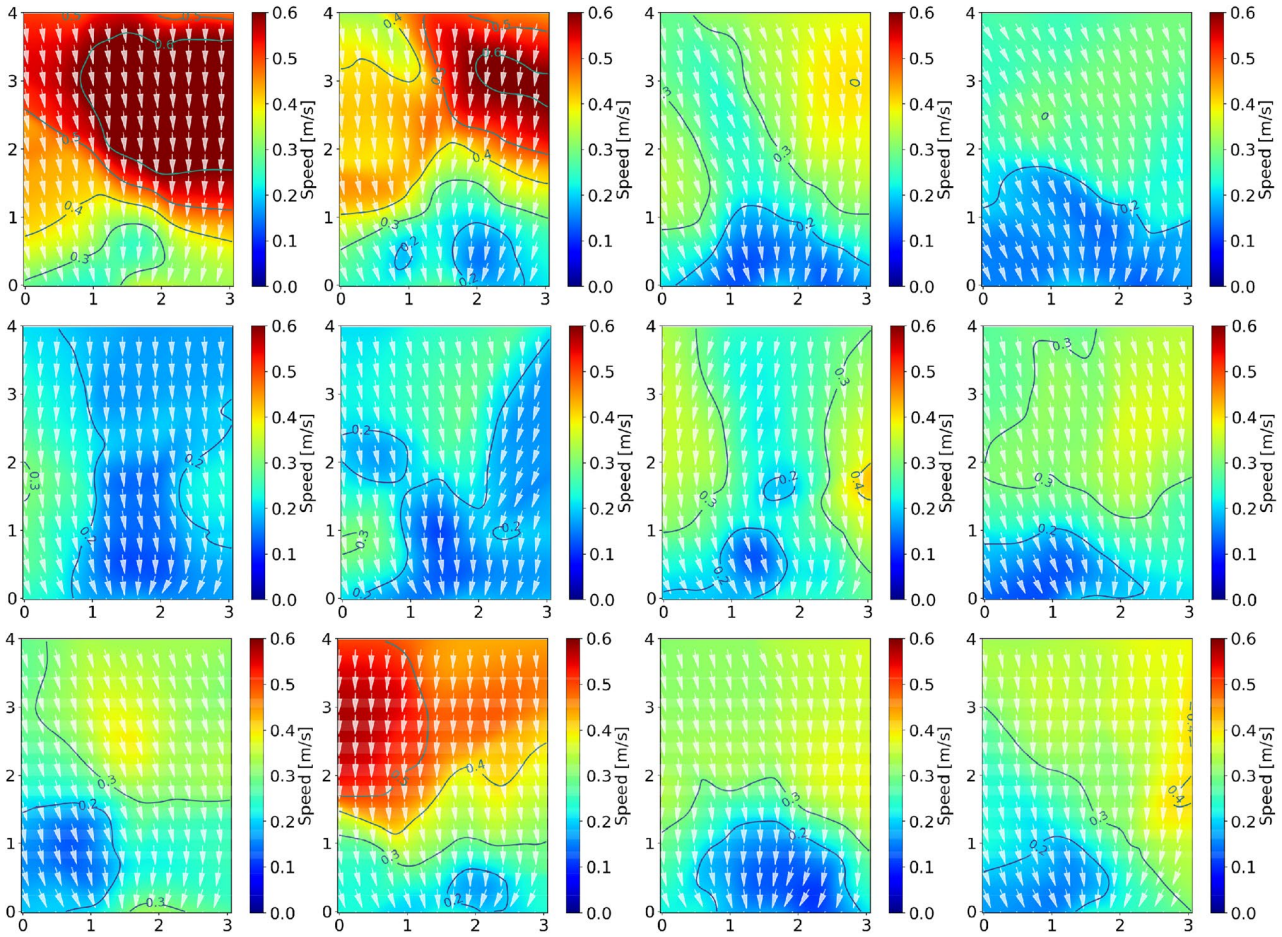
Velocity profiles during the congested stage in this study. Top row: r1, r2, r3, r4; middle row: r5, r6, r7, r8; bottom row: r9, r10, r11, r12. The color represents the value of the velocity and the white arrows represent the direction of the velocity. The dark blue lines represent the speed contours.

**Figure 7:**
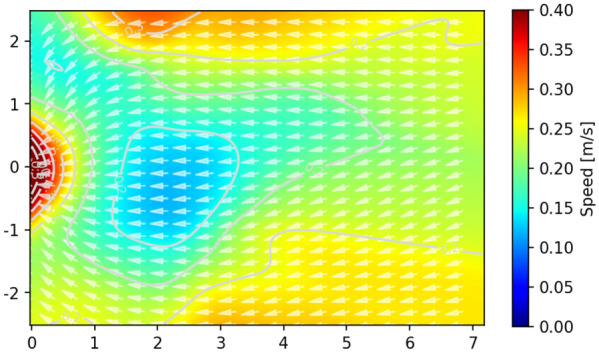
Velocity profiles during the congested state for highly motivated students’ bottleneck experiment (b = 1.05 m)^8^. Color for the speed value, white arrows for the speed directions, and grey lines for speed contours. Note that the bottleneck length (1.9 m) is omitted to better compare with the velocity map of children’s experiments, as we focus on the velocity inside the artificial room.

We further study the distribution of speed in the congested state (the congested state). The speed profiles are calculated based on the method mentioned in “Local density and local speed” section and shown in Figs. 6 and 7. The direction of velocity is calculated as Eq. (4).

4 $$e_{{x_{t} }} = \frac{{(x_{t + 12} - x_{t - 12} )/25.0}}{N}, \;e_{{y_{t} }} = \frac{{(y_{t + 12} - y_{t - 12} )/25.0}}{N}, \;N = \sqrt {v_{{x_{t} }}^{2} + v_{{y_{t} }}^{2} }$$

In the children’s experiments, the speed in front of the exit is smaller than that in other regions and the speed is around 0.1 m/s due to the congestion around the exit. Similarly, in students’ velocity profiles, the value of the lowest speed region is also around 0.1 m/s. However, the location of the lowest speed region is near the exit in the children’s experiment while the speed region locates around 2 m away from the bottleneck entrance in the students’ experiment. Unlike children, the students accelerate when they reach near the bottleneck entrance (about 0.5 m in front of the exit), forming an arch-like higher speed distribution. The phenomenon is influenced by the peak density region locating in the front of the bottleneck entrance described in “Density profiles” section.

5 $$angle = \arctan \left( {v_{j} /v_{i} } \right)$$

*v*_*i*_ and *v*_*j*_ represent the component of the velocity in the direction perpendicular and parallel to the exit, respectively.

**Figure 8:**
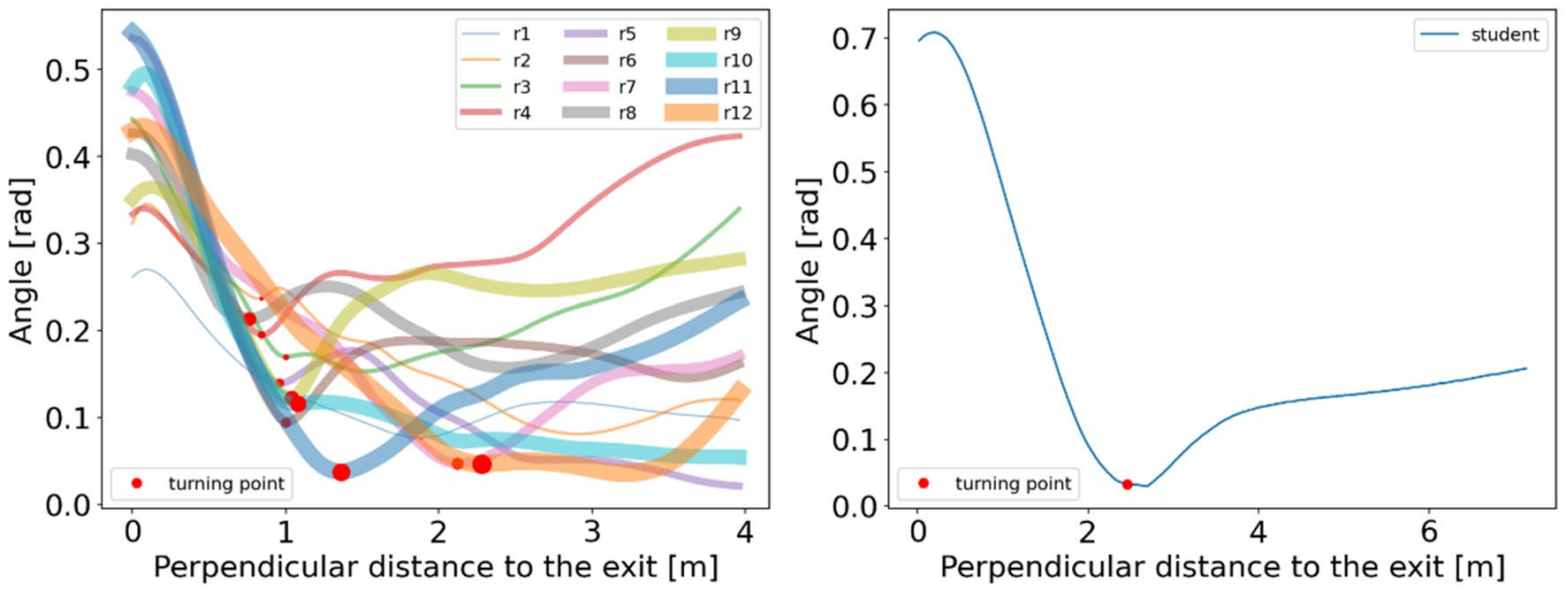
Relation between the movement direction and the distance perpendicular to the exit in children’s (Left) and students’ (Right) experiment. The colored lines represent the evolution of movement direction with the perpendicular distance. The red circles represent that pedestrians change their movement directions from the relatively smooth variation state to the rapid state. In the Left figure, to distinguish the experimental run, the linewidth and the size of the circles are adjusted.

To quantify the movement direction of pedestrians, the angle of movement direction is defined as Eq. (5). Figure 8 shows that the moving direction keeps a relatively smaller value at the beginning of the experiment and increases to higher values when the perpendicular distance to the exit is smaller than a threshold value. For children, the median and mean values of the threshold are 1.02 m and 1.26 m, combined with all experimental runs. However, for students, they change their directions towards the exit at 2.46 m distance perpendicular to the exit, which is earlier than that of children. Interestingly, combined with the threshold value and the density profiles in Fig. 5, we observe that the threshold value obtained in this section corresponds to the 5 ped/m^2^ region both for children and for adults. The phenomenon indicates that pedestrians (both children and adults) choose to decrease the perpendicular distance to the exit at first and start to change their movement direction when they reach the high-density region (about 5 ped/m^2^).

## Characteristic time

### Motion activation time

To measure the motion activation ability of pedestrians, the time taken from having enough space to start moving is defined as motion activation time *t*_*MA*_ and computed for each pedestrian in each experimental run applying the method presented in “Motion activation time” section

#### Distribution

**Figure 9:**
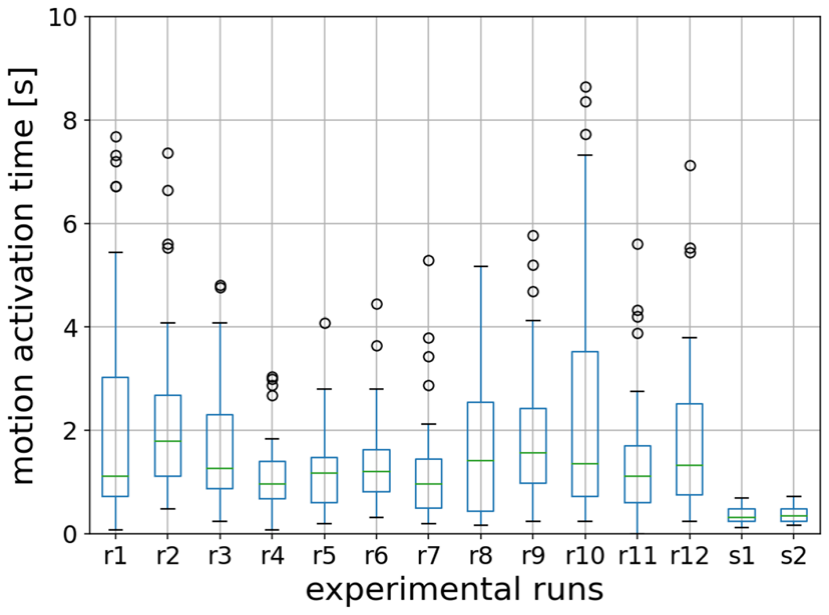
The distribution of motion activation time of children and university students in different experimental runs. *r* for the experimental run of children’s experiment and *s* for that of students’. The circles represent the outliers.

Figure 9 shows the overall distribution of motion activation time of children and students in each experimental run. Scheffe’s test is applied to analyze the difference between multiple groups of motion activation time. The results show that there is no significant difference between different runs, which indicates that the repetitions of the experiment show no significant improvement in reducing the motion activation time of children in this study, which is contrary to presupposition. Besides, we observe that children spend more time (around 1 s) to move compared to adults (around 0.3 s).

**Figure 10:**
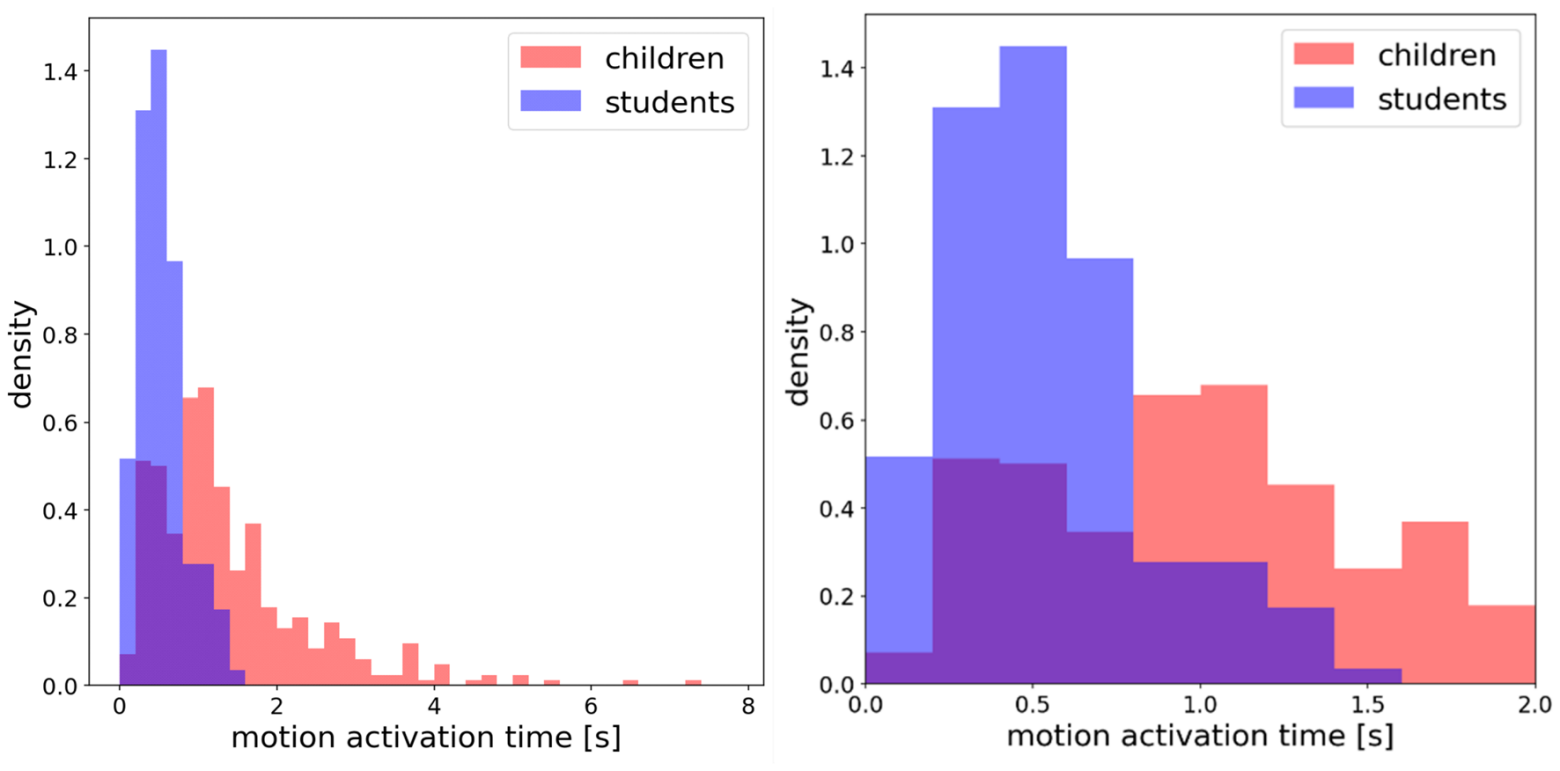
Left: Probability density histogram of motion activation time of children (blue) and adults (red) overall. Right: Detailed histogram of motion activation time in the range of 0–2 s. The bin size is set as 0.2 s.

To obtain the distribution pattern of the motion activation time, the outliers are removed to exclude the effects. Figure 10 shows the probability density histogram of children and students. The skewness for children and students is 1.81 and 0.91, respectively. The motion activation time is right-skewed both for children and for students. The kurtosis of children and students is 4.91 and 0.52, respectively. The data sets of children with higher kurtosis imply that it tends to have heavy tails or outliers, compared to that of students. This indicates that children are more likely to have longer motion activation time, compared to that of students. With the similar experimental setup and the same vocal signal, we infer that the longer motion activation time of children is mainly due to the easily distracted preschool children compared to that of students. The further empirical studies are needed to verify the assumption.

As most data distribute between 0–2 s, Fig. 10 right is plotted to detailed analyze the difference between the motion activation time of children and adults. The motion activation time of students concentrated around 0.2–0.4 s, while the children’s data distribute much scattered in the range of 0.2–1.2 s. The motion activation time of students is rather concentrated compared to that of children. The individual difference in motion activation time between students is smaller than that of children.

Based on the above analysis, Erlang distribution is applied to fit the data. The probability distribution function of Erlang distribution is.

$$f\left( {x;k;\mu } \right) = \frac{{x^{k - 1} e^{{ - \frac{x}{\mu }}} }}{{\mu^{k} \left( {k - 1} \right)!}} for x,\quad \mu \ge 0$$.

**Figure 11:**
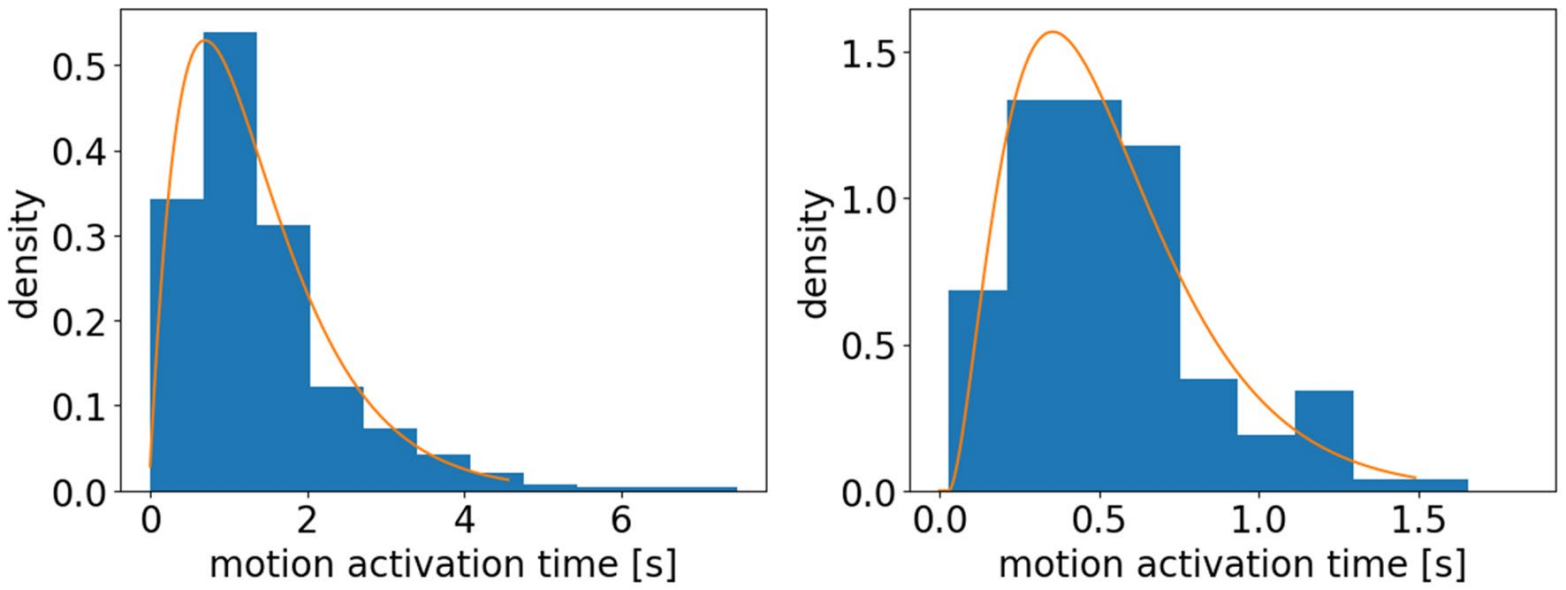
Probability density function (PDF) of the motion activation time for children (left) and students (right). Erlang distribution fits the data well.

The fitting results are shown in Table 3. Erlang distribution fits well both for children’s data and for students’ data (see Fig. 11). Based on the fitting results, the probability density of motion activation time is single-peak distributed both for children and for adults. The values of peak density are 0.53 and 1.57 and the corresponding motion activation time is 0.70 s and 0.36 s for children and students, respectively.

**Table 3:** Fitting results of the distribution of motion activation time of children and students.

	*k*	*μ*	*Adj.R*^*2*^
Children	2.057	0.679	0.961
Students	2.797	0.181	0.921

**Figure 12:**
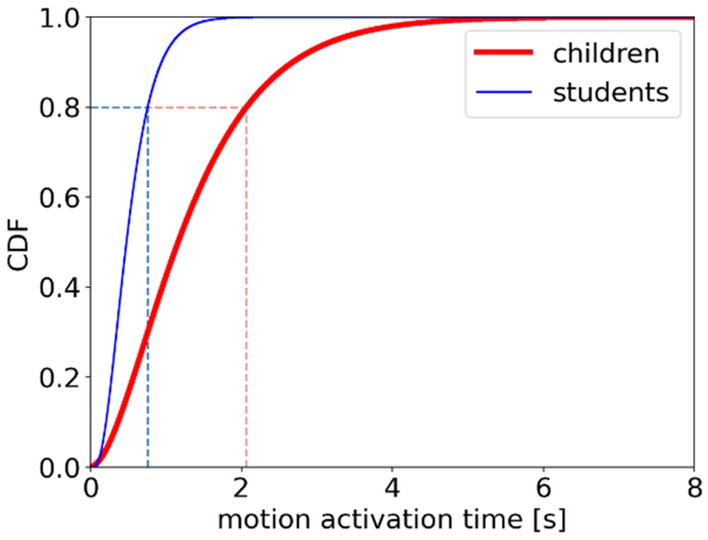
Cumulative distribution function of motion activation time of children (red thick line) and students (blue fine line).

Cumulative distribution function (CDF) is investigated to better obtain the distribution of motion activation time (Fig. 12). The escalating trend of students is more rapidly compared to that of children. 80% of children and students have a motion activation time lower than 2.07 s and 0.76 s, respectively. The above analyses indicate that the motion activation time of children is higher than that of students and the distribution of motion activation time is scattered of children and concentrated of students.

#### Influence of initial position

**Figure 13:**
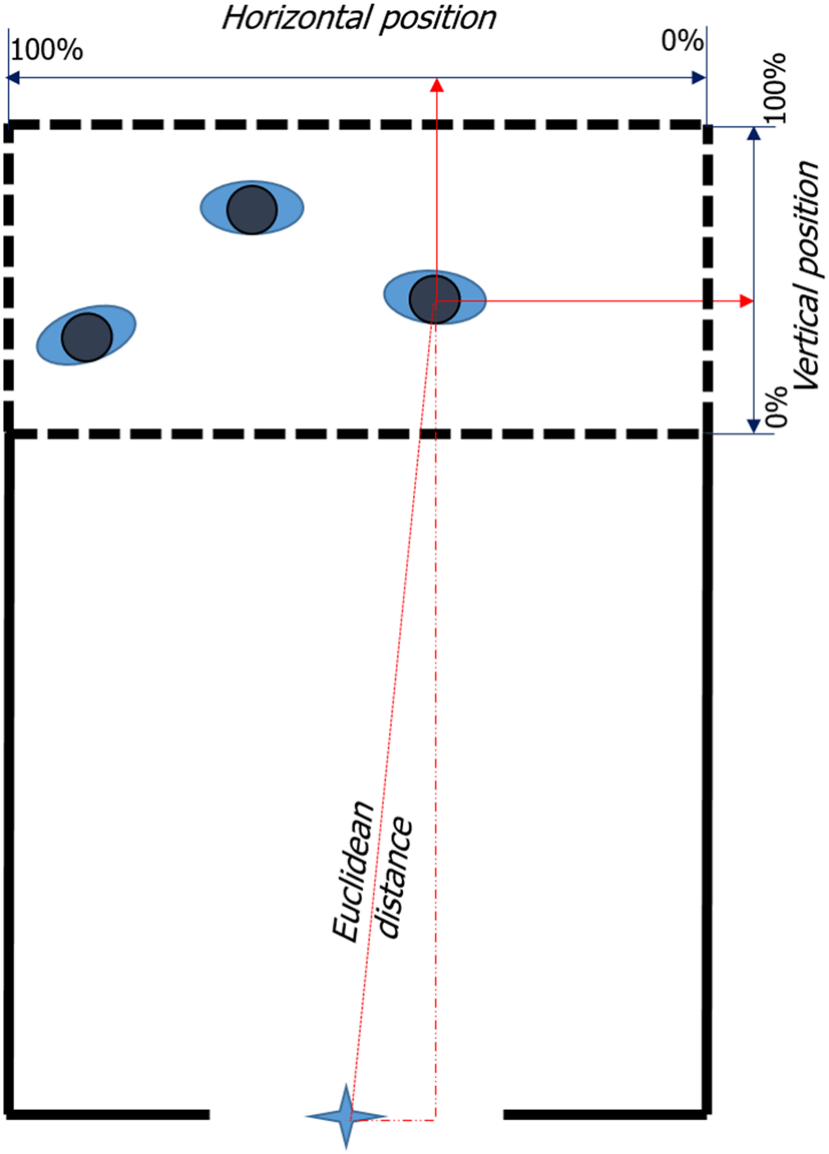
Definition of vertical and horizontal position. The blue star represents the middle point of the exit. Pedestrian stand in the waiting area (dashed area) initially, waiting for the vocal signal to start the experiment. The values of the intersections of solid red lines and light blue axes are the vertical and horizontal positions of the pedestrian.

Besides the distribution, the influencing factors on the motion activation time are further investigated. Initial positions of pedestrians in the crowd are considered and the position is divided into horizontal and vertical positions to quantify their influence on the motion activation time. The horizontal and vertical position is calculated based on the definition shown in Fig. 13.

**Figure 14:**
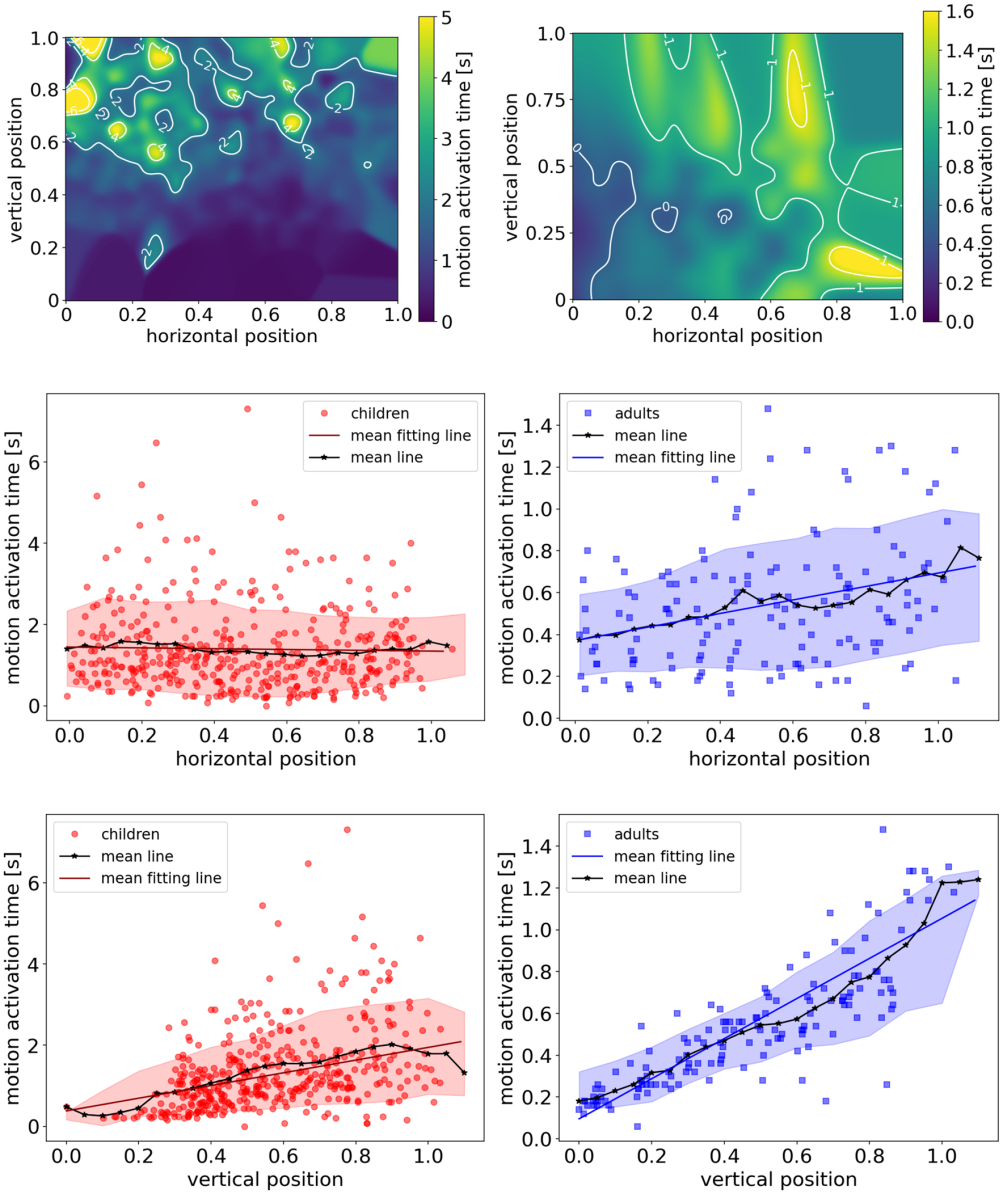
The influence of horizontal and vertical position on the motion activation time of pre-school children (red points) and adults (blue squares). Top row: spatial distribution in children’s (left) and adults’ (right) experiment. Middle row: for the horizontal position. Bottom row: for vertical position. Left column: for pre-school children. Right column: for adults. The black stars are the mean values of horizontal/vertical position for each bin and the colored bands give the standard error. The mean data (black stars) are obtained as the result of a vertical binning procedure^30^ over a large number of measurements. The bin size is set as 0.05. The black lines highlight the mean data trend and the red/blue lines show the linear fitting results. Note that some points are located beyond 100%, which is due to that some pedestrians stand outside the waiting area.

Based on Fig. 14 top rows, we observe that the motion activation time shows an increasing trend with the increasing vertical position while no obvious trend with the increasing horizontal position. To test the hypothesis, mean data of vertical binning procedure^30^ are calculated and linear regression fitting is applied to the mean data. The fitting results are shown in Table 4 and Fig. 14 middle and bottom row. The fitting results show that there is a linear relation between motion activation time and vertical position. However, there is no obvious relation between motion activation time and horizontal position based on the linear regression method.

**Table 4:** Linear regression fitting results of the relation between horizontal/vertical position to the motion activation time of children and students.

Linear regression	Scatter			Mean		
	*k*	*b*	*R*^*2*^	*k*	*b*	*R*^*2*^
**Horizontal distance**						
Children	− 0.21	1.48	0.00	− 0.21	1.49	0.31
Students	0.30	0.38	0.08	0.31	0.38	0.83
**Vertical distance**						
Children	1.90	0.30	0.15	1.77	0.31	0.91
Students	0.89	0.11	0.70	0.94	0.10	0.94

To verify this, the Pearson correlation method is applied to test the relation between motion activation time and horizontal/vertical position, and the results are shown in Table 5. Between motion activation time and horizontal position, there is no correlation for that of children and a weak correlation for that of students. However, between motion activation time and vertical distance, there is a moderate correlation for that of children and a strong correlation for that of students. Except for the correlation between the horizontal position and motion activation time of children, other correlation results are all significant (see Sig. in Table 5). The correlation results verify the fitting result in Fig. 14. Vertical distance influences the motion activation time both for children and for adults, while horizontal distance shows no obvious impact.

**Table 5:** Correlation results between motion activation time and horizontal/vertical position of children and students.

Pearson correlation	Children		Students	
	Horizontal position	Vertical position	Horizontal position	Vertical position
**Motion activation time**				
Correlation coefficient	− 0.054	0.390	0.292	0.839
Sig. (two-tailed)	0.27	1.11E–16*	0.00036*	1.35E–39*

*at 0.01 level (two-tailed), the result is significant.

Based on the above discussion, we obtain that the vertical position shows a significant impact on the motion activation time both for children and for students. Focusing on the fitting slope and Fig. 14, children’s motion activation time is more sensitive to the vertical position compared to that of students. Children who stand in front of the crowds react much quickly compared to the children in the back, while the phenomenon is less obvious for students.

### Relaxation time

Relaxation time is investigated to measure the speed adjustment capability of children and students during the whole experiment. The relaxation time is defined as the time taken to increase speed by 0.1 m/s. See “Relaxation time” section for a detailed definition. The distribution and impact factors are considered in this section.

#### Distribution

**Figure 15:**
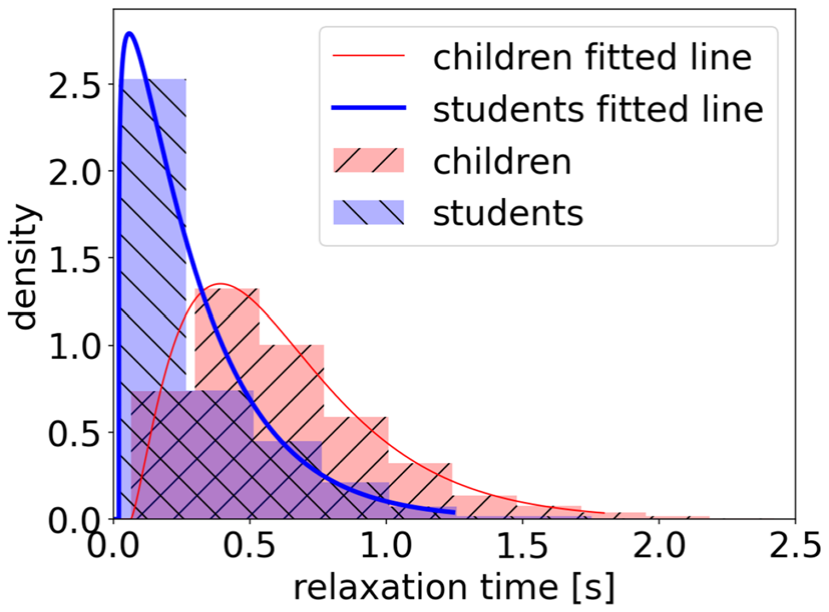
Probability density function (PDF) of the relaxation time for children (red) and students (blue). An Erlang distribution fits the data well.

Applying the method described in “Relaxation time” section, we obtain the probability density distribution of the relaxation time of children and students (Fig. 15). The relaxation time of students is smaller than that of children, indicating that students spend less time to accelerate compared to that of children. As the experimental setup is similar, we deduce that the difference in the relaxation time between children and students is mainly due to the immature physical properties of children.

The skewness for children and students is 1.15 and 1.89, respectively. The relaxation time is right-skewed both for children and for students. The kurtosis of children and students is 1.54 and 4.60, respectively. The data sets of students with higher kurtosis imply that it tends to have heavy tails or outliers. The distributions of children adults are not a normal distribution. Erlang distribution is applied to fit the data. The fitting results are listed in Table 6. Erlang distribution fits the data well and the probability density is single-peak distributed, both for children and students. The values of peak density are 1.35 and 2.79 and the corresponding relaxation time is 0.39 s and 0.06 s for children and students, respectively. This indicates that a large proportion of students could adjust their speed by 0.1 m/s in 0.06 s, while a large percentage of children have to spend 0.39 s to improve the same speed. We should notice that the speed range is 0–2 m/s for children and 0–4 m/s for adults.

**Table 6:** Fitting results of the distribution of relaxation time of children and students.

	*k*	*μ*	*Adj.R*^*2*^
Children	2.39	0.24	0.998
Students	1.15	0.25	0.974

**Figure 16:**
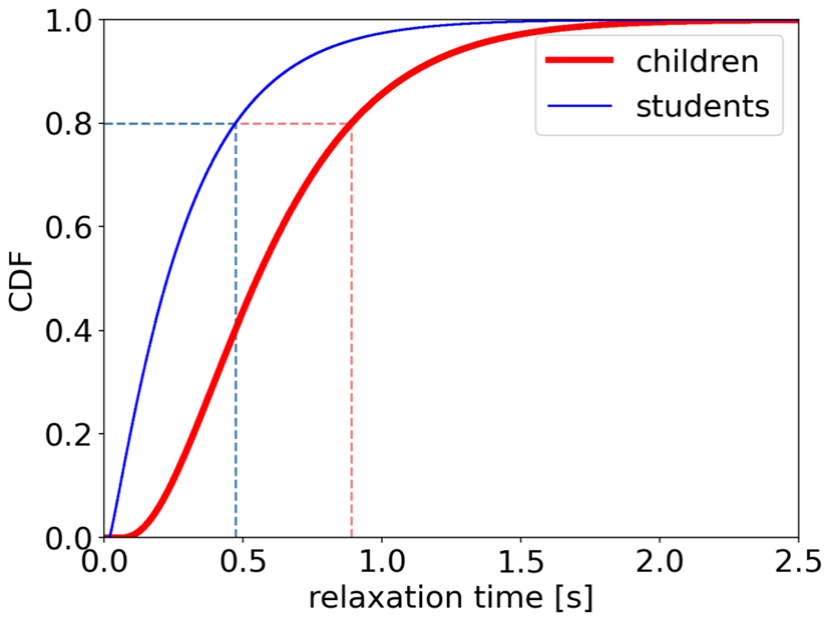
Cumulative distribution function of relaxation time of children (red thick line) and students (blue fine line).

Besides the PDF of the relaxation time, the cumulative distribution function (CDF) is investigated to better obtain the distribution of relaxation time as shown in Fig. 16. The escalating trend of students is more rapidly compared to that of children. 80% of students could adjust their speed by improving 0.1 m/s in 0.47 s, while 80% of children have to spend 0.89 s to improve the same speed. The above analyses indicate that the students adjust their speeds more quickly compared to that of children.

#### Impact of initial speed on the relaxation time

**Figure 17:**
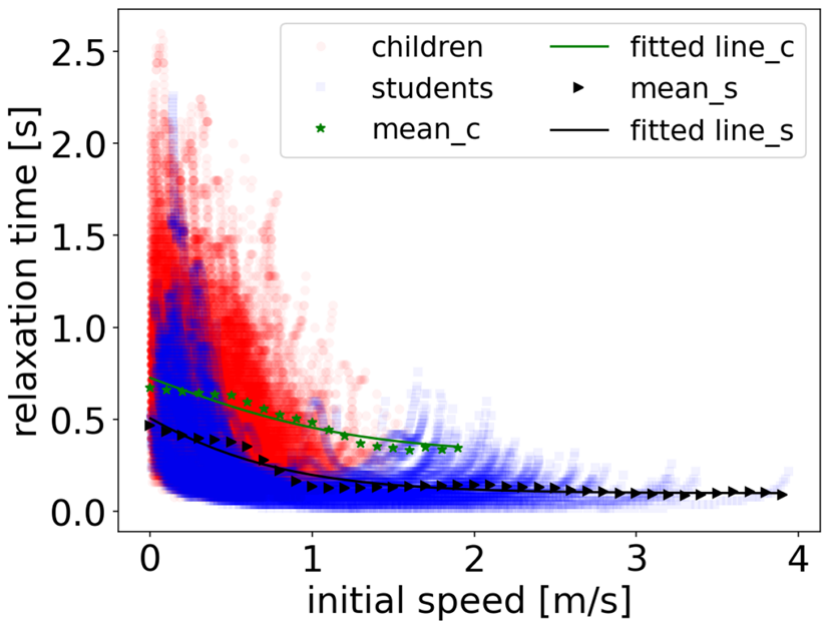
The influence of initial speed on the relaxation time of children (red circles) and students (blue circles). The green stars and black triangles represent the mean data of children and students by applying vertical binning procedure^30^, respectively. The bin size is set as 0.1 m/s. The green and black lines highlight the fitting results of children and students, respectively.

To further measure the acceleration ability of pedestrians during the whole movement, we explore the influence of initial speed on the relaxation time (see Fig. 17). With the increasing initial speed, the relaxation time shows a decreasing trend, representing that pedestrians require a shorter time to accelerate compared to that for lower initial speed. Besides, children spend more time accelerating than that of students.

**Figure 18:**
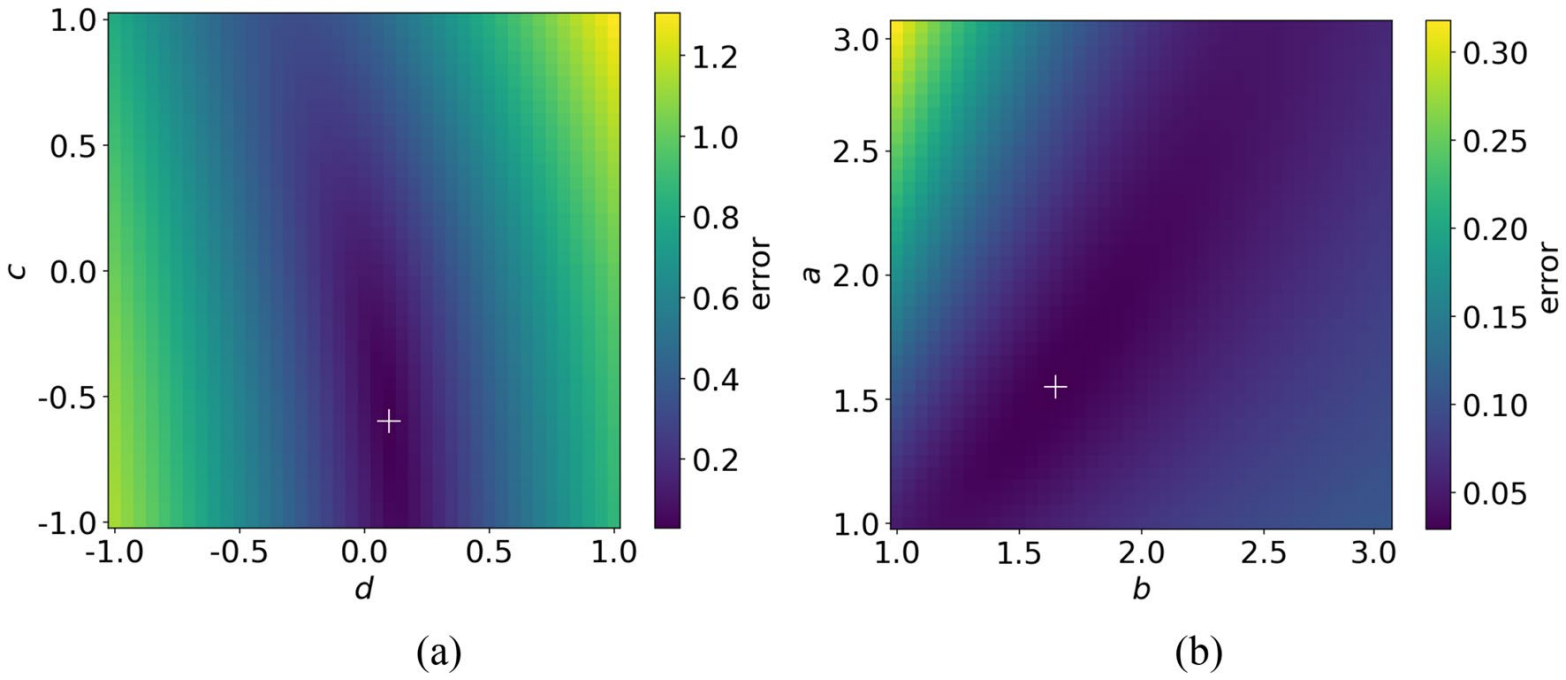
Effect of the model parameters *a, b, c, d* on the error between model results and the experimental results. In (**a**), *c* and *d* are fixed as  − 0.62 and 0.10. In (**b**), *a* and *b* are fixed as 1.54 and 1.65. The white cross represents the position of minimum error.

To measure the trend quantitatively and to compare between children and students, mean data is obtained by applying vertical binning procedure^30^. We observe that students adjust the speed more quickly than that of children. Focusing on the data of students, with the increasing speed, the relaxation time first declines and then stabilizes around a value when the initial speed exceeds a threshold value. To quantify this, the sigmoid function (Eq. (6) is employed to fit the trend and the fitting results are listed in Table 7. The fitting results are obtained by applying the cross-entropy method (CE method)^31^. Besides, an exhaust algorithm is applied to test whether the fitting results of the model parameters are local optimums (see Fig. 18). The data of students’ experiments are tested and the results show that it is feasible to calculate the parameters of the model using the CE method.

**Table 7:** Fitting results of the relation between relaxation time and initial speed of children and students.

	*a*	*b*	*c*	*D*	Adj.R^2^
Children	0.86	1.52	− 0.04	0.31	0.92
Students	1.54	1.65	− 0.62	0.10	0.93

Focusing on the fitting result in Fig. 17, when the initial speed of students exceeds 1.03 m/s, the corresponding relaxation time oscillates around 0.11 s. We assume that there is a similar trend in children’s data. Based on the fitted sigmoid function, we assume that when the initial speed exceeds 2.36 m/s for children, the relaxation time oscillates around 0.33 s. Further research needs to be performed to verify the assumption.

6 $$y = \frac{a}{{1 + e^{{b\left( {x - c} \right)}} }} + d$$

### Time gap

To measure the dynamics of pedestrian flow when passing through the exit, the time gap is analyzed in this section. The time gap is defined as the time elapsed between two consecutive pedestrians passing through the exit.

**Figure 19:**
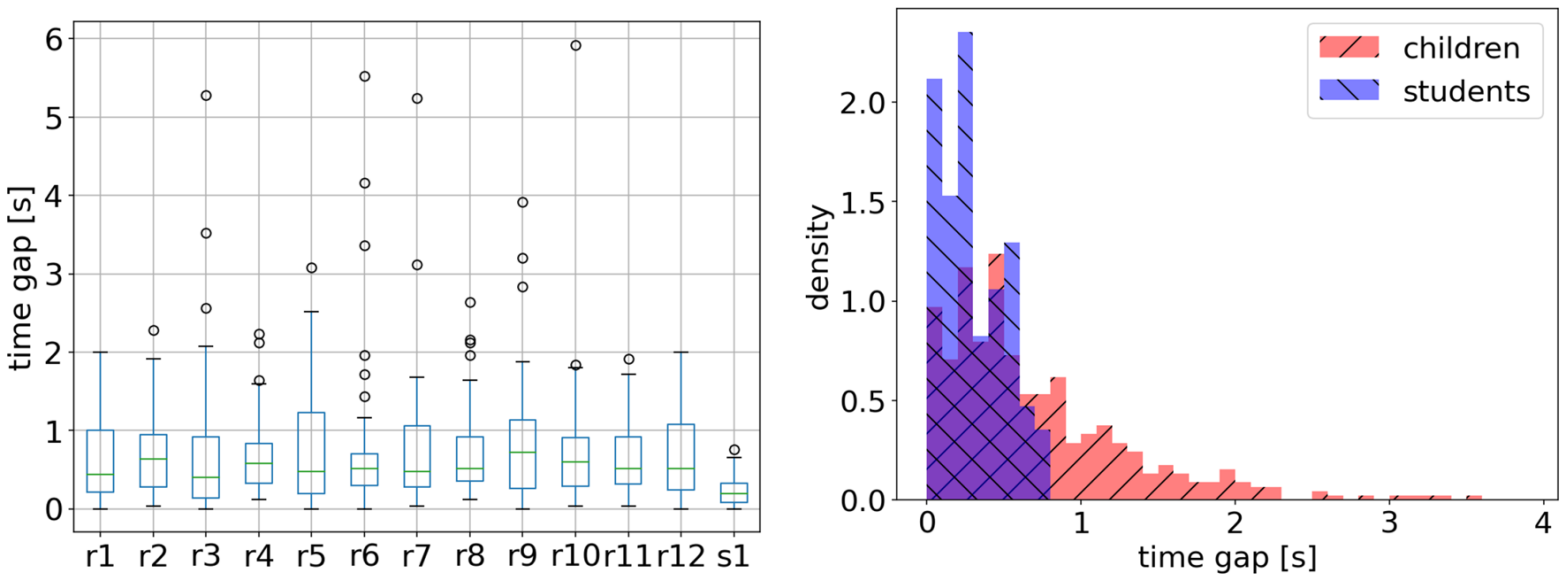
Distribution of time gap of children and students. Left: boxplot of data in each experimental run, r for children’s and s for students’ experiment. Right: probability density histogram, red for children, and blue for students.

Based on the boxplot of the time gap (see Fig. 19), we observe that the time gap of children is higher than that of students and the time gap shows no significant change with the experiment repetition, which is different from the previous study stating that repetitions help reduce the evacuation time^32^. The mean time gap of children and students is 0.75 s and 0.29 s; the median value is 0.52 s and 0.26 s for that of children and students, respectively. The difference between children’s and students’ time gap is significant (one-sample t test: sig = 1.05E–7). The shorter time gap of students indicates that the flow of students is much smoother than that of children. Although both children and students were asked to leave the artificial room as if in a fire emergency, the competition of children was more fiercely than that of students based on the recorded videos. The fierce competition shows a negative impact on the traffic efficiency of the exit, resulting in a long time gap of children.

To study the distribution of the time gap, the skewness for children and adults is 2.89 and 0.48, respectively. The relaxation time is right-skewed both for children and for students, while students’ data is less skewed. The kurtosis of children and students is 12.40 and  − 0.86, respectively. The data sets of children’s time gap with higher kurtosis imply that it tends to have heavy tails or outliers, indicating that a longer time gap prefers to appear in children’s experiments. The negative value of the kurtosis of students implies that the distribution is similar to the uniform distribution. Unlike children’s data, the time gap of students is more regular, distributing more uniform between 0 s and 0.76 s in this study.

### Travel time inside the exit

Travel time inside the exit *tt*_*exit*_ is considered to measure the traffic efficiency near the exit. To investigate whether the position when entering the exit region shows an impact on the travel time, the incident angle is defined and considered. Initial Voronoi density in the exit region is considered to measure the degree of crowdedness on traffic efficiency. Detailed definition of *tt*_*exit*_, incident angle *α,* and initial Voronoi density can be seen in “Relaxation time” section.

Based on the results of SPSS^33^ (see Table 8), there is no relation between the incident angle and *tt*_*exit*_. Different from our assumption, the entering position shows no obvious impact on *tt*_*exit*_ both for children and for students in this study. We suspect that as pedestrians gather around the exit and form arch-like distribution, pedestrians who enter the exit region face a similar congested condition despite the entering position, resulting in a similar *tt*_*exit*_.

**Table 8:** Correlation results between travel time and incident angle/initial Voronoi density in the exit region of children’s and students’ experiments.

Pearson correlation	Children		Students	
	Incident angle	Initial Voronoi density	Incident angle	Initial Voronoi density
**tt**_**exit**_				
Correlation coefficient	0.124	0.678	0.105	0.928
Sig. (two-tailed)	0.007*	8.26E–65*	0.152	6.84E–38*

*at 0.01 level (two-tailed), the result is significant.

**Figure 20:**
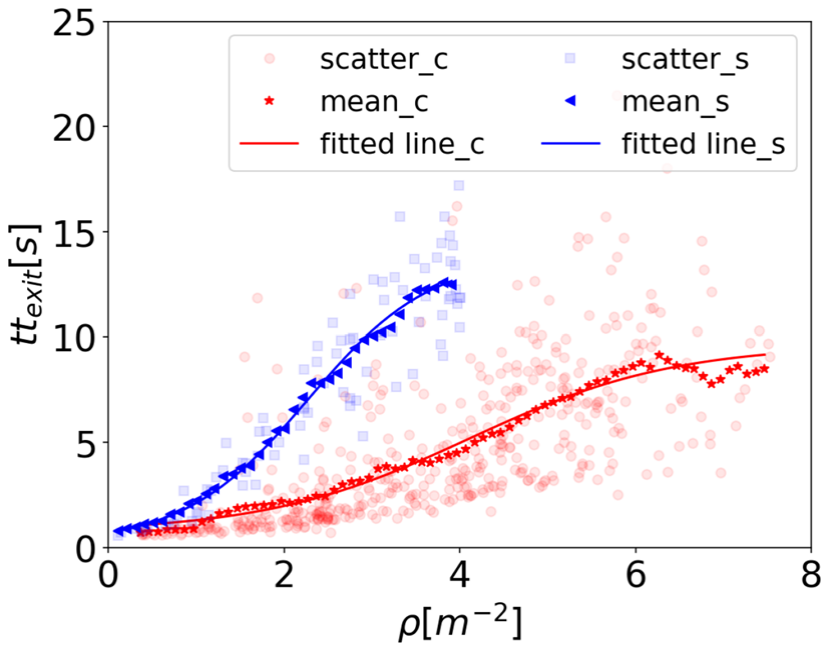
Relation between travel time inside the exit region *tt*_*exit*_ and initial Voronoi density in the exit region for children (red points) and students (blue squares). Red stars and blue triangles for the mean data of children and of students, respectively. Red and blue lines highlight the fitting results of the trend.

Based on the Pearson correlation results, initial Voronoi density in the exit region shows a strong impact on the travel time both for children and for students. To further quantify the influence and obtain the difference between children and adults, the relation between initial Voronoi density and *tt*_*exit*_ is analyzed in Fig. 20.

*tt*_*exit*_ increases with the increasing initial Voronoi density in the exit region as we expected. The sigmoid function is applied to fit the data and the results show that it fits well. The function is $y = \frac{a}{{1 + e^{{b\left( {x - c} \right)}} }} + d$ (same as Eq. 5). The fitting results are listed in Table 9. Based on the fitting results, we assume that there exists a density threshold that when the density exceeds the value, the travel time is stable around a constant value. The threshold value of density and *tt*_*exit*_ for children is 11.8 ped/m^2^ and 9.63 s, and for adults is 7.60 ped/m^2^ and 13.86 s, respectively. To verify the assumption, a further experimental study is needed in the future.

**Table 9:** Fitting results of the relation between relaxation time and initial speed of children and students.

	*a*	*b*	*c*	*D*	Adj.R^2^
Children	8.92	− 0.86	4.11	0.72	0.982
Students	13.70	− 1.42	2.27	0.17	0.996

## Summary

In this study, we investigate the movement characteristics of children passing through an exit and compare with that of students. Density profiles, velocity profiles, characteristic time are analyzed to obtain the movement characteristics and differences of children and students. By introducing the motion activation time, we measure the movement characteristics of pedestrians at the beginning of the movement process. Relaxation time helps quantify the speed adjustment ability of children and students.

Density profiles and velocity profiles reveal the macro feature of pedestrians of high motivated children and students around the bottleneck during the congested state, point out that the distributions of density are inhomogeneous both for children and for adults and the density is arch-like distribution. Besides, motion activation time, relaxation time highlight that students adjust the movement more quickly and much smoother than that of children in the whole process of the movement. The difference in the time gap shows that children compete more fiercely than students around the bottleneck.

The findings in this study further demonstrate that high motivated pedestrians prefer to gather around the exit and the density is arch-like distribution. The definition of motion activation time in this study helps quantify the movement characteristics in the initialization phase of pedestrians and measure the difference between children and students. The results can provide empirical data to help validate and verify the model, like the probability density function of motion activation time and relaxation time.

Further researches should take advantage of the information gained from this study to develop algorithms for simulating the movement of pedestrians by combing their physical characteristics and mobility. Further data must be collected on the motion activation time and relaxation time. These are key elements in determining the motion ability of pedestrians and further obtain the evacuation time of pedestrians. These findings, and the suggested work, will have an important impact on enhancing the understanding of the difference between children and adults, future code/regulatory changes, engineering guidance.

## Supplementary Information

Supplementary Information.
