# Supplementary material for: Characteristic time in highly motivated movements of children and adults through bottlenecks
Source: Sci Rep. 2021 Mar 3;11:5096. doi: 10.1038/s41598-021-84324-4 (PMC7930252; doi:10.1038/s41598-021-84324-4)
Supplement: Supplementary file 1 — Supplementary Information. [file 41598_2021_84324_MOESM1_ESM.pdf]

# Characteristic time in highly motivated movements of children and adults through bottlenecks

Hongliu Li[1,2], Jun Zhang[1*], Long Xia[1], Libing Yang[3], Weiguo Song[1], Kwok Kit Richard Yuen[2]
1. State Key Laboratory of Fire Science, University of Science and Technology of China, Jinzhai Road 96, Hefei, Anhui, People's Republic of China
2. Department of Architecture and Civil Engineering, City University of Hong Kong, Kowloon 999077, Hong Kong, People's Republic of China
3. College of Civil Engineering and Architecture, Hunan Institute of Science and Technology, Xueyuan Road, Yueyang, Hunan, People's Republic of China
*Corresponding author's email: junz@ustc.edu.cn

# Appendix A. The selection of 1.05 m width bottleneck experiment of students.

In this study, we aim to investigate the similarity and difference of movement characteristics of pre-school children and university adults running through bottlenecks. Table. A1 shows the shoulder breadth information of children and adults based on the *Human Dimensions of Chinese Minors (GB/T 26158-2010)* and *Human Dimensions of Chinese Adults (GB/T 10000-1988).*

Table. A1 Selected value of shoulder breadth of pre-school children [1] and adults [2].

| People | Shoulder breadth/m | |
|---|---|---|
| | Male | Female |
| Children (median value) | 0.286 | 0.282 |
| Adults (18-25 years old)(90% value) | 0.454 | 0.415 |

The mean height of the male students and female students is 1.76 m and 1.62 m, respectively [3]. Based on the standard [2], 90% male Chinese and female aged 18-25 years owns a height lower than 1.764 m and 1.647 m. Considering this, the shoulder width is selected as 0.454 m and 0.415 m, meaning that 90% shoulder width of 18-25 years old Chinese is smaller than the values. Considering the gender ratio of the participants, the shoulder width of pre-school children and students is set as 0.284 m and 0.435 m, respectively.

Based on the shoulder breadth of pre-school children and of 18-25 years-old adults and the 0.7 m width bottleneck experiment of children, a 1.05 m-width bottleneck experiment of students [3] is selected to compare the movement characteristics passing

through the bottleneck between children and adults.

## Appendix B. The comparison between raw trajectories and smoothed trajectories of children and adults.

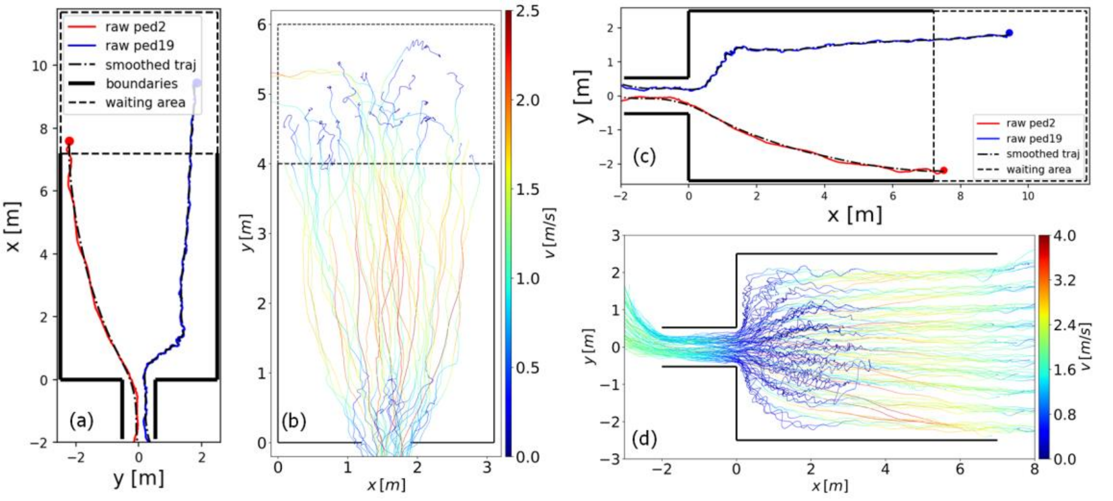


Fig. B1 Comparison between raw trajectories and smoothed trajectories of children and adults. Typical trajectories (raw and smoothed) in the children's experiment (a) and in the adults' experiment (c). The raw trajectories with the colored speed in children's experiment (b) and in adults' experiment (d).

Based on the illustrated trajectories, the raw trajectories oscillate more severe in the waiting area and in the front of the bottleneck both for children and for adults. The oscillations appear due to the swaying heads of pedestrians in low speed. Qualitatively, the oscillations are more severe of adults' trajectories compared with that of children's. Fig. B1(b) and B1(d) show the trajectories and speeds of children and of adults, respectively. The colored speed information verifies that the oscillations around the bottleneck entrance and in the waiting area are resulted from low speed. Pedestrians sway their heads when they shift weight at low speed, resulting into oscillating trajectories, especially in front of the bottleneck.

*Fréchet Distance*[4] is applied to measure the similarity between smoothed trajectories and raw trajectories as well as to quantify the degree of heads' swaying. Fig. B2 shows the obtained *Fréchet Distance* between raw and smoothed trajectories of children and of adults. For all repetitions of the children's experiment, the calculated distance is lower than that in adults' experiment. The qualitative results verify that the adults' trajectories oscillate much severe compared to that of preschool children.

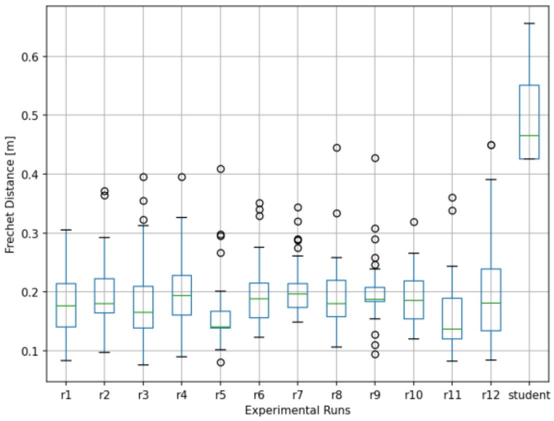


Fig. B2 *Fréchet Distance* between raw trajectories and smoothed trajectories of children and of adults.

# Appendix C. Time profiles of density and speed in the measurement area

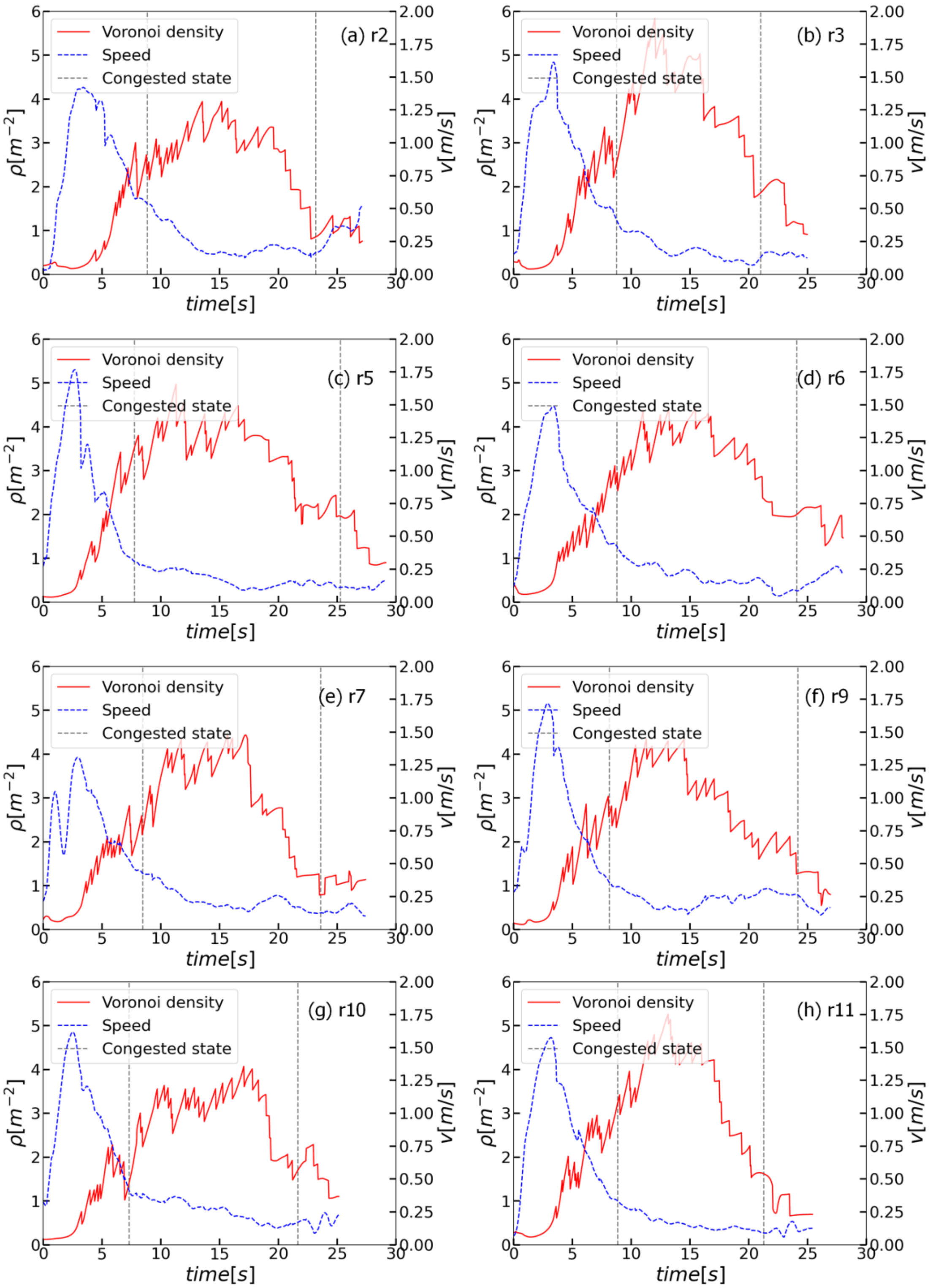


Fig. C1 Time profiles of density and speed in the measurement area in different runs of children's experiment. Red solid lines represent the time profiles of density and blue dashed lines represent the time profiles of speed. The range between grey dashed lines represent the selected congested state.

# Appendix D. Range of density and speed in the congested state in

the measurement area.

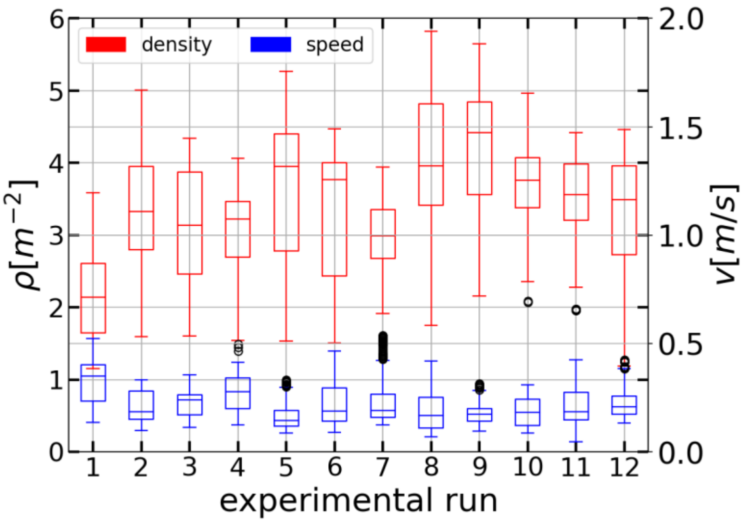


Fig. D1 Boxplot of density (red) and speed (blue) in the measurement area in the congested state. The density ranges from around 2 ped/$m^2$ to 6 ped/$m^2$. The speed ranges from 0 m/s to 0.5 m/s.

# Appendix E Preschool children's motivation during the experiment.

In the experiment, we asked the participants to leave the artificial room quickly as if in fire and the participants said that they understood the experiment. During the experiment, the preschool children moved under the command of their tutors and they passed the bottleneck quickly with high motivation. As shown in Fig. E1, the speeds of children are higher than 1.5 m/s before the bottleneck (about 1.0 m away from the exit) and after passing the bottleneck. The speed near the exit is lower than 1.0 m/s is due to the restriction of the bottleneck and the high-density regions. Without the restriction of the bottleneck, the children accelerate and run at a high speed. The phenomenon shows that children were high-motivated during the experiment. When in a real emergency, the pre-school children are also high-motivated and under the command of their tutors. Considering this, the experimental results can reflect the motion characteristics of preschool children in a real emergency to some extent.

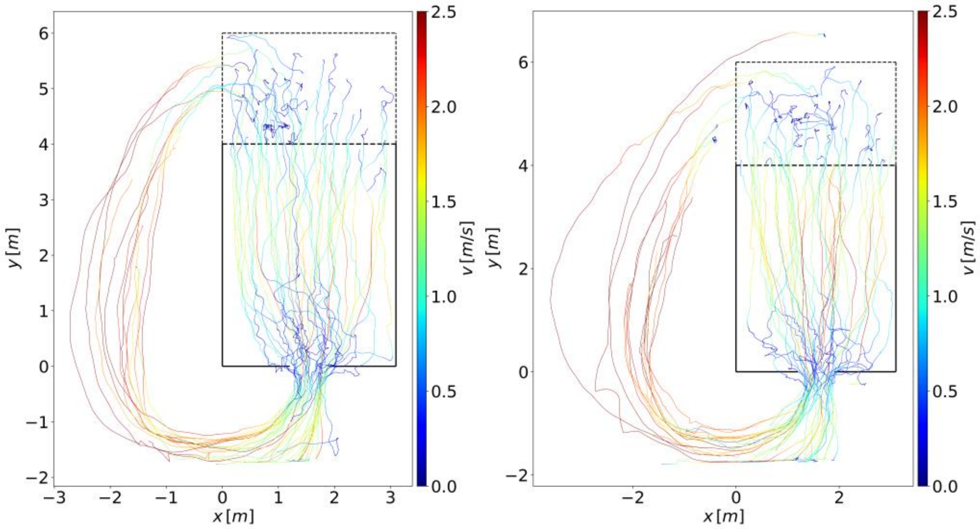


Fig. E1 Raw trajectories of preschool children passing through the bottleneck. Left: r7. Right: r10. The color represents the speed of preschool children.

## References

1 Supervision, C. S. B. o. T. Vol. GB/T 26158-2010 (China Standard Press, 2011).

2 Supervision, C. S. B. o. T. Vol. GB/T 10000-1988 17 (China Standard Press, 1988).

3 Li, H., Zhang, J., Song, W. & Yuen, K. K. R. A comparative study on the bottleneck pedestrian flow under different movement motivations. *Fire Safety Journal*, 103014, doi:https://doi.org/10.1016/j.firesaf.2020.103014 (2020).

4 Eiter, T. & Mannila, H. Computing discrete Fréchet distance. (Citeseer, 1994).
